# Axillary and gut microbiota characteristics in axillary bromhidrosis patients and the effect of microwave therapy: a case-control study

**DOI:** 10.3389/fmicb.2026.1769465

**Published:** 2026-05-05

**Authors:** Xia Wu, Mingxi Li, Yaohan Xu, Xinyu Liu, Zhou Gao, Xiaoyun Jiang, Jianping He, Yaping Wu, Chongming Wu

**Affiliations:** 1 Department of Dermatology, Sir Run Run Shaw Hospital, Zhejiang University School of Medicine, Hangzhou, Zhejiang, China; 2 School of Chinese Materia Medica, Tianjin University of Traditional Chinese Medicine, Tianjin, China; 3 Yuncheng County Hospital of Traditional Chinese Medicine, Yuncheng, Shandong, China; 4 Tianjin Key Laboratory of Therapeutic Substance of Traditional Chinese Medicine, Tianjin, China; 5 State Key Laboratory of Chinese Medicine Modernization, Tianjin, China

**Keywords:** axillary bromhidrosis, axillary microbiota, correlation analysis, gut microbiota, microwave therapy, the gut-skin axis

## Abstract

**Introduction:**

Axillary bromhidrosis is characterized by excessive sweat gland activity and foul body odor, significantly affecting patients’ psychological well-being and social interactions.

**Methods:**

This study recruited 30 axillary bromhidrosis patients and 30 healthy controls, collecting sweat and stool samples for microbiome analysis. Among the patients, 8 patients received microwave therapy, collecting pre- and post-treatment samples for microbiome analysis.

**Results:**

The axillary microbiota of patients showed significant differences compared to healthy controls, particularly with increased abundance of odor-causing bacteria such as *Staphylococcus* and related species (*Staphylococcus hominis*, *Staphylococcus haemolyticus*) (LDA > 3, *p* < 0.05). While the gut microbiota composition showed no significant changes, but LEfSe analysis revealed that SCFAs-producing bacteria (*Bacteroides stercoris*, *Phocaeicola massiliensis*, and *Phocaeicola vulgatus*) was significantly elevated (LDA > 3, *p* < 0.05), indicating that the abundance changes of SCFAs-producing bacteria may be associated with axillary odor production through the regulation of metabolic processes. Correlation analysis revealed positive correlations between axillary odor-producing genera (*Staphylococcus*, *Peptoniphilus*, *Anaerococcus*) and gut SCFAs-producing genera (*Roseburia*, *Blautia*, *Clostridium*), suggesting a bidirectional microbiota network through gut-derived butyrate production and immune modulation (*p* < 0.05). However, further experimentals are required to confirm the causal relationship. Furthermore, microwave therapy significantly altered axillary microbiota diversity, potentially alleviating axillary odor by inhibiting odor-producing bacteria (*Staphylococcus*) (LDA > 3, *p* < 0.05), while exerting minimal impact on the gut microbiota. KEGG pathway enrichment analysis revealed significant metabolic activity changes in lipid, carbohydrate, sulfur, and amino acid metabolism pathways.

**Conclusion:**

This study is the first to demonstrate the interrelationship between the axillary and gut microbiota in axillary bromhidrosis patients, showing a link between gut-derived butyrate production and axillary odor. These findings offer new insights into the microbiological mechanisms underlying axillary bromhidrosis and identify potential microbial targets for future gut-based systemic treatments.

## Graphical abstract

**Figure:**
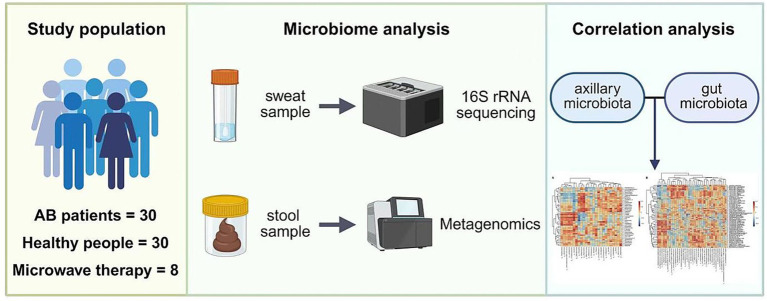

## Introduction

Axillary bromhidrosis is characterized by acidic and volatile odors produced by microbial metabolism secretions from the apocrine glands in the axilla. These offensive odors often leave a negative impression during social interactions, leading to significant psychological stress and adversely affecting social life. Studies have shown that the prevalence of axillary bromhidrosis is approximately 6% in the Chinese population, while it reaches as high as 90% in Caucasians and Africans (Zhang et al., 2024). Current treatment primarily focus on reducing sweat gland secretion and axillary bacterial load, including topical medications, botulinum toxin injections, laser therapy, microwave therapy, and surgery. While surgery is effective in severe cases, it is frequently associated with postoperative complications such as skin necrosis, scarring, bleeding, nerve damage, or infection (He et al., 2012). In contrast, microwave therapy offers the advantages of being minimally invasive, scar-free, minimal pain, rapid recovery, and low complication rates, making it a minimally invasive alternative to surgery (Hsu et al., 2017).

The development of axillary bromhidrosis involves multiple factors, including genetic background, sweat gland secretion, and the microbiota. The axillary region is rich in apocrine, eccrine, and sebaceous glands, which provide a nutrient-rich environment for microbial communities (Kim et al., 2021). Research has identified resident genera in the axilla, such as *Staphylococcus*, *Micrococcus*, *Corynebacterium*, and *Propionibacterium*, with *Staphylococcus hominis* and *Corynebacterium tuberculostearicum* being considered key contributors to axillary odor (Callewaert et al., 2017). These axillary microbiota utilize specific enzymatic pathways to convert odorless sweat precursors into odorous compounds, such as sulfur-containing aliphatic alcohols, volatile short-chain fatty acids, and steroid derivatives (Fredrich et al., 2013). For instance, Watanabe et al. highlighted that *Staphylococcus hominis* is significantly more abundant in axillary bromhidrosis with a strong, cumin-like odor (type C) compared to the milder, milk-like odor (type M). This bacterium contributes to the production of the major odorant in axillary bromhidrosis, 3-methyl-3-sulfanylhexan-1-ol (3M3SH). Notably, the PatB (MalY) enzyme of *Staphylococcus hominis* exhibits high catalytic efficiency in the biosynthesis of 3M3SH (Watanabe et al., 2024). These findings provide a crucial theoretical foundation for the development of targeted microbiome-based odor control strategies.

In recent years, the concept of the “gut-skin axis” has drawn increasing attention to the relationship between the gut microbiota and skin health. The human microbiome consists of approximately 10 trillion microorganisms, primarily located on the body surface (skin microbiota) and within the body (gut and oral microbiota) (Sender et al., 2016; Liu et al., 2026). The skin and gut share high functional and structural similarities; both are highly innervated and vascularized organs with complex immune systems, neuroendocrine regulatory mechanisms, and diverse microbial communities (De Pessemier et al., 2021). The gut microbiota regulates systemic immunity, endocrine, and nervous systems through metabolic products such as short-chain fatty acids (SCFAs) and neurotransmitters. Dysbiosis of the gut microbiota can increase host vulnerability, disrupt mucosal immune tolerance, affect skin barrier function, and alter local microbial balance (Mahmud et al., 2022; Jimenez-Sanchez et al., 2025; Yang et al., 2025). Numerous studies have linked gut dysbiosis to skin diseases such as atopic dermatitis, acne, and psoriasis (Salem et al., 2018). For instance, hidradenitis suppurativa (HS), a chronic inflammatory skin disease commonly occurring in the axillary region, is characterized by apocrine gland obstruction, secondary bacterial infections, and associated odor and pain. A clinical study found elevated levels of *Ruminococcus gnavus* and *Clostridium ramosum* in the gut microbiota of HS patients, along with enrichment of metabolic pathways related to D-glucuronic acid degradation and D-galacturonic acid degradation, which are associated with Crohn’s disease (CD) (McCarthy et al., 2022). These findings suggest a close functional link between the gut and skin. Furthermore, probiotics targeting the gut microbiota have shown potential in promoting skin health. For example, mice fed *Lactobacillus reuteri* exhibited improved epidermal thickness and increased hair follicle generation, resulting in shinier and thicker fur (Erdman and Poutahidis, 2014). Another placebo-controlled clinical study revealed that oral probiotics reduced skin sensitivity and enhanced skin barrier recovery (Gueniche et al., 2014). Notably, oral probiotics have also demonstrated therapeutic potential in treating eczema, atopic dermatitis, acne, skin allergies, UV-induced skin damage, and wound healing (Gao et al., 2023; Parhizkar et al., 2025). Further research into the correlation between gut and skin microbiota may provide new insights into the role of specific gut microbiota in skin diseases, particularly in the underexplored field of axillary bromhidrosis.

Although the relationship between the axillary microbiota and body odor is well-established, and the systemic impact of the gut microbiota on skin health has been widely reported, the potential association between axillary and gut microbiota in axillary bromhidrosis remains unexplored. To address this gap, we collected sweat and stool samples from 30 axillary bromhidrosis patients and 30 healthy controls. And 8 patients subsequently received microwave therapy, collecting pre- and post-treatment samples for 16S rRNA sequencing and metagenomic analysis. Our analysis identified differential microbes in sweat and stool associated with axillary bromhidrosis and explored correlations between the axillary and gut microbiota. These findings not only deepens the understanding of axillary bromhidrosis pathogenesis, but also offers new insights into gut-based systemic therapeutic strategies.

## Method

### Study population

This study randomly recruited 30 patients with axillary bromhidrosis (AB) and 30 healthy controls (Con) from Sir Run Run Shaw Hospital, Zhejiang University School of Medicine, between March 2023 and March 2024. All participants were of Han ethnicity and had not received any clinical treatments related to this study prior to enrollment. Inclusion criteria were: clinically diagnosed with bilateral axillary bromhidrosis, meeting the diagnostic criteria for axillary bromhidrosis outlined in the Chinese Clinical Dermatology; complete clinical data; and informed consent. Exclusion criteria included: any skin disease or other conditions that could affect the results; chronic diarrhea; prior antibiotic use before stool sample collection. Additionally, to investigate the impact of microwave therapy on the axillary and gut microbiota, we collected sweat and stool samples from 8 axillary bromhidrosis patients who underwent a single microwave therapy session, with samples collected before treatment and 3 months post-treatment for sequencing analysis. Specifically, the inclusion criteria were as follows: clear symptoms of axillary bromhidrosis; no prior treatment for the condition; willingness to participate in the study and provide informed consent. Furthermore, these 8 patients covered a range of axillary bromhidrosis severity, including 3 with severe, 3 with moderate, and 2 with mild forms, to investigate the broad applicability and generalizability of microwave therapy. At least 12 h prior to sweat sample collection, participants refrained from showering or using any hygiene products (soap, cosmetics, deodorants, or antiperspirants). After remaining stationary for 30 min, sterile cotton swabs were used to collect samples from a 50 cm^2^ area of each axilla for 3 min. The swabs were immediately placed in preservation tubes, rapidly frozen in liquid nitrogen, and stored at −80 °C for subsequent 16S rRNA sequencing. Participants also self-collected at least 3 g of fresh stool in sterile containers, which were frozen in liquid nitrogen and stored at −80 °C for metagenomic analysis. All studies involving human subjects adhered to the World Medical Association’s Ethical Guidelines (Helsinki Declaration), received approval from the Ethics Committee of Sir Run Run Shaw Hospital, Zhejiang University School of Medicine (ethical approval number: 20250954), and informed consent was acquired from all research participants.

### Sweat 16S rRNA sequencing

Genomic DNA was extracted from sweat samples using a DNA kit and evaluated via 1% agarose gel electrophoresis. The V3–V4 region of the 16S rRNA gene was PCR-amplified using genomic DNA as a template. The amplicons were purified, quantified, and used to construct sequencing libraries by Biozeron Biotechnology (Shanghai). Sequencing was performed on the Illumina MiSeq PE300 platform. Raw data quality was assessed using FastQC, and low-quality sequences, primers, and adapters were removed. Further annotation was performed using QIIME2, generating an operational taxonomic unit (OTU) table. OTUs were classified based on 97% similarity using the RDP classifier and the SILVA138 reference database. Rarefaction analysis was performed to ensure data representativeness and comparability.

### Stool metagenomics

Genomic DNA was extracted from stool samples using a DNA kit, with quality evaluated by 1% agarose gel electrophoresis and DNA concentration and purity measured. PCR products were purified, and sequencing libraries were constructed by Biozeron Biotechnology (Shanghai). Shotgun sequencing was performed using the Illumina NovaSeq 6,000 system with 5 Gb depth. Trimmomatic was used to remove adapter sequences, low-quality reads, high-N sequences, and short reads. Sequences were assembled into contigs using Megahit, followed by gene prediction using METAProdigal and clustering with CD-HIT to create a non-redundant gene set. The predicted protein sequences were annotated by aligning with NR, eggNOG, KEGG, CARD, CAZy, and other databases.

### Bioinformatics analysis

Bioinformatics analyses included diversity, composition, and differential species analysis. Alpha diversity was assessed using the Chao1, Shannon, and Simpson indices. Beta diversity was analyzed through principal coordinate analysis (PCoA) based on Bray-Curtis distance. The composition of the gut microbiota was analyzed at different taxonomic levels. Wilcoxon rank-sum tests were used to compare microbial composition between groups, with the Benjamini–Hochberg method applied to control the false discovery rate (FDR) at <0.05. KEGG pathway enrichment analysis was performed to identify potential biological pathways and functions associated with the targets. Data analysis and visualization were performed using R packages vegan (V 2.6.2) and ggplot2 (V 3.5.1).

## Results

### Study design and clinical characteristics of participants

This study recruited 60 participants, including 30 axillary bromhidrosis patients (14 males, 16 females) and 30 healthy controls (5 males, 25 females). The average age of patients was 30.23 years, with a mean BMI of 22.43. The healthy controls had an average age of 33.27 years, with a mean BMI of 21.93. Both groups were comparable in baseline characteristics, consistent with the study’s paired design. Additionally, the patients included 13 with mild (43%), 11 with moderate (37%), and 6 with severe (20%) cases, reflecting the clinical diversity of axillary bromhidrosis and enhancing the external validity of the results. Detailed information for all participants are provided in Supplementary Table S1, and baseline characteristics of all participants are listed in Supplementary Table S2.

### Changes in axillary microbiota composition and structure in axillary bromhidrosis patients

**Figure 1:**
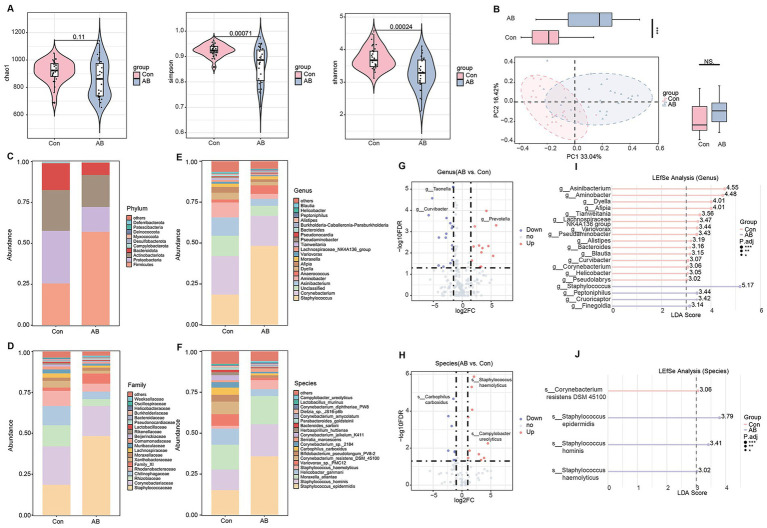
Axillary microbiota profiles of axillary bromhidrosis patients and healthy controls. **(A)** α-diversity indices (Chao1, Simpson, Shannon). **(B)** Principal coordinate analysis (PCoA). **(C–F)** Taxonomic composition at the phylum, family, genus, and species levels. **(G)** Volcano plot of differential genera. **(H)** Volcano plot of differential species. **(I)** LEfSe analysis at the genus level. **(J)** LEfSe analysis at the species level. *FDR < 0.05, **FDR < 0.01, ***FDR < 0.001 indicate significant differences versus healthy controls.

The axillary microbiota is closely associated with the development of axillary bromhidrosis. To explore changes in the axillary microbiota composition and structure, we collected sweat samples from patients and healthy controls for 16S rRNA analysis. Compared to the Con group, the AB group showed significantly lower Shannon and Simpson indices (*p* < 0.001), with a non-significant decrease in the Chao1 index (Figure 1A). PCoA analysis revealed a significant difference in the overall microbiota structure between the groups along the PC1 axis (*p* < 0.001), while no significant difference was found along the PC2 axis (Figure 1B). At the phylum level, the relative abundance of Firmicutes was significantly higher, and Proteobacteria was significantly lower in the AB group (*p* < 0.001) (Figure 1C). At the family level, Staphylococcaceae was significantly more abundant, and Rhizobiaceae was significantly less abundant in the AB group (*p* < 0.001) (Figure 1D). At the genus level, *Staphylococcus* was significantly more abundant, while *Asinibacterium* was significantly less abundant in the AB group (*p* < 0.001) (Figure 1E). At the species level, *Staphylococcus epidermidis* and *Staphylococcus hominis* had significantly higher relative abundances in the AB group (*p* < 0.001), while *Helicobacter ganmani* was slightly lower (Figure 1F). Volcano plots at the genus and species levels identified 30 and 19 differentially abundant taxa, respectively, with 17 genera and 9 species downregulated, and 13 genera and 10 species upregulated in the AB group (relative abundance > 0.1%, FDR < 0.05) (Figures 1G,H). LEfSe analysis further revealed that the AB group was enriched in *Staphylococcus*, *Peptoniphilus*, *Cruoricaptor*, and *Finegoldia* at the genus level (Figure 1I), and in *Staphylococcus epidermidis*, *Staphylococcus hominis*, and *Staphylococcus haemolyticus* at the species level (LDA > 3, *p* < 0.05) (Figure 1J).

### Changes in gut microbiota composition and structure in axillary bromhidrosis patients

**Figure 2:**
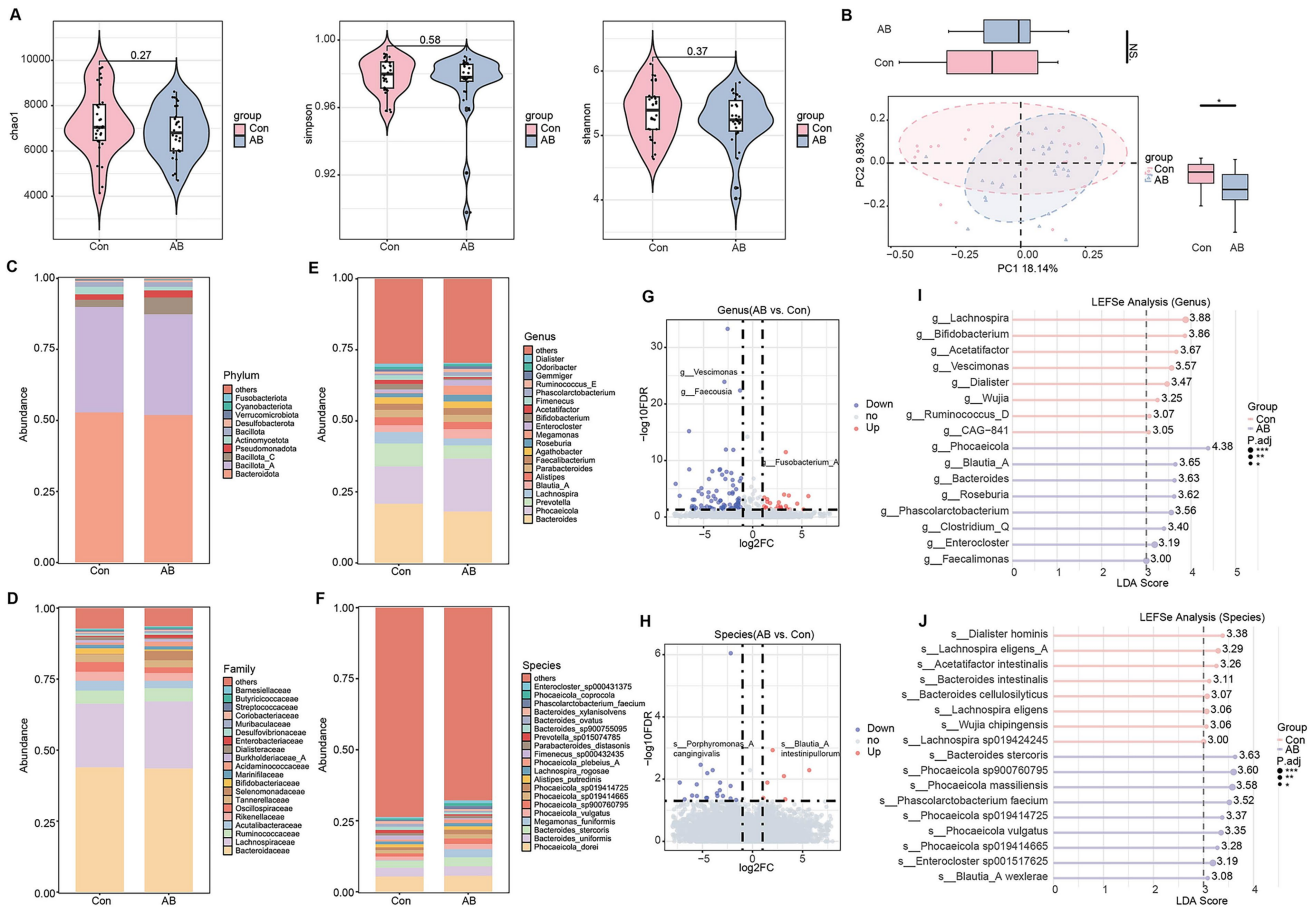
Gut microbiota profiles of axillary bromhidrosis patients and healthy controls. **(A)** α-diversity indices (Chao1, Simpson, Shannon). **(B)** Principal coordinate analysis (PCoA). **(C–F)** Taxonomic composition at the phylum, family, genus, and species levels. **(G)** Volcano plot of differential genera. **(H)** Volcano plot of differential species. **(I)** LEfSe analysis at the genus level. **(J)** LEfSe analysis at the species level. *FDR < 0.05, **FDR < 0.01, ***FDR < 0.001 indicate significant differences versus healthy controls.

The gut microbiota is closely associated with skin diseases. To explore changes in the gut microbiota composition and structure, we collected stool samples from patients and healthy controls for metagenomic analysis. Alpha diversity analysis showed no significant differences in Chao1, Simpson, or Shannon indices between the two groups (Figure 2A). PCoA analysis revealed a significant difference in the overall microbiota structure between the groups along the PC2 axis (*p* < 0.05), while no significant difference was found along the PC1 axis (Figure 2B). At the phylum level, the relative abundance of Bacillota_C was significantly higher (*p* < 0.05), and Actinomycetota was slightly lower in the AB group compared to the Con group (Figure 2C). At the family level, Selenomonadaceae was slightly more abundant, and Oscillospiraceae was significantly less abundant in the AB group (*p* < 0.05) (Figure 2D). At the genus level, *Phocaeicola* was significantly more abundant (*p* < 0.05), and *Prevotella* was slightly less abundant in the AB group (Figure 2E). At the species level, *Megamonas funiformis* had a slightly higher relative abundance in the AB group (Figure 2F). Volcano plots at the genus and species levels identified 115 and 28 differentially abundant taxa, respectively, with 90 genera and 20 species downregulated, and 25 genera and 8 species upregulated in the AB group (relative abundance > 0.1%, FDR < 0.05) (Figures 2G,H). LEfSe analysis further revealed that the AB group was enriched in *Phocaeicola*, *Blautia_A*, *Bacteroides*, and *Roseburia* at the genus level (Figure 2I), and in *Bacteroides stercoris*, *Phocaeicola massiliensis*, *Phascolarctobacterium faecium*, and *Phocaeicola vulgatus* at the species level (LDA > 3, p < 0.05) (Figure 2J).

### Correlation analysis between axillary and gut microbiota

**Figure 3:**
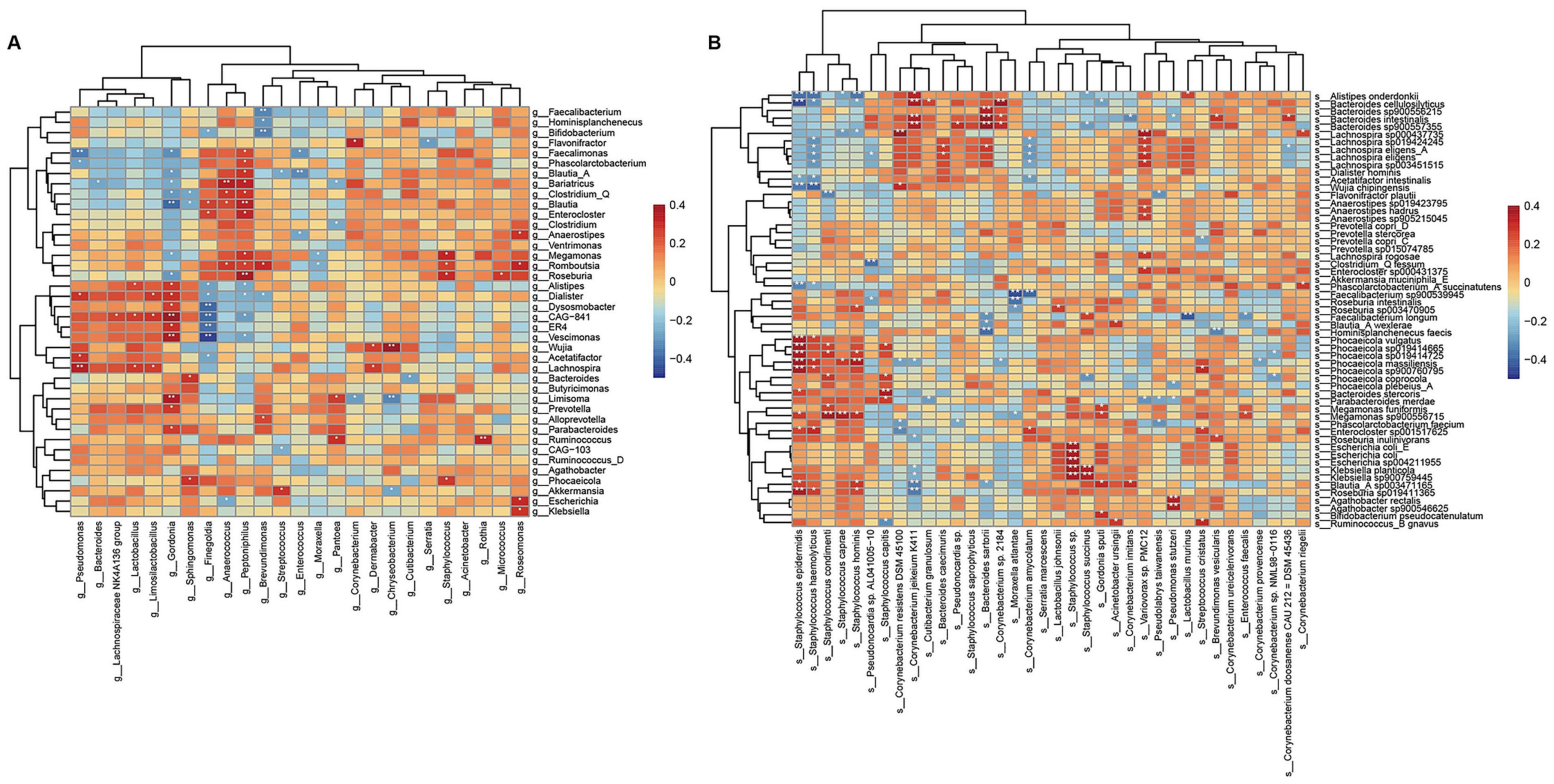
Correlation analysis of differential axillary and gut microbiota between axillary bromhidrosis patients and healthy controls: **(A)** genus-level correlation heatmap and **(B)** species-level correlation heatmap.

To explore the relationship between the axillary and gut microbiota in axillary bromhidrosis patients, we performed Spearman correlation analysis on the differential taxa. First, in the axillary differential genera, *Lactobacillus* was significantly negatively correlated with *Finegoldia*, *Anaerococcus*, and *Peptoniphilus*, while a significant positively correlation with *Bacteroides*, *Lachnospiraceae NK4A136 group*, and *Limosilactobacillus* (*p* < 0.01) (Supplementary Figure S1A). Among the axillary differential species, *Staphylococcus haemolyticus* and other odor-producing species were significantly positively correlated with *Serratia marcescens*, and negatively correlated with *Corynebacterium jeikeium K411* and *Corynebacterium resistens DSM 45100* (*p* < 0.01) (Supplementary Figure S1B). In the differential genera of the gut microbiota, *Blautia* was significantly positively correlated with *Roseburia*, *Faecalimonas*, and *Enterocloster*, and negatively correlated with *Alistipes*, *Butyricimonas*, and *Prevotella* (*p* < 0.01) (Supplementary Figure S2A). In the differential species of the gut microbiota, *Escherichia coli* was significantly positively correlated with *Klebsiella planticola* and *Anaerostipes hadrus*, and negatively correlated with *Bacteroides intestinalis* and *Phocaeicola coprocola* (*p* < 0.01) (Supplementary Figure S2B). Finally, correlation analysis between the axillary and gut microbiota revealed that *Staphylococcus* and *Peptoniphilus* were significantly positively correlated with *Megamonas*, *Romboutsia*, and *Roseburia* (*p* < 0.05), while *Anaerococcus* and *Peptoniphilus* were positively correlated with *Blautia*, *Romboutsia*, and *Clostridium* (*p* < 0.05). Interestingly, *Gordonia* was positively correlated with *Alistipes* and *Prevotella* (*p* < 0.05), while *Finegoldia* showed the opposite pattern (Figure 3A). At the species level, *Staphylococcus haemolyticus* and other *Staphylococcus* species were positively correlated with *Bacteroides stercoris*, *Escherichia coli*, *Phocaeicola vulgatus*, and *Bifidobacterium pseudocatenulatum*, and negatively correlated with *Alistipes onderdonkii* and *Lachnospira eligens* (Figure 3B).

### Changes in axillary and gut microbiota after microwave therapy in axillary bromhidrosis patients

**Figure 4:**
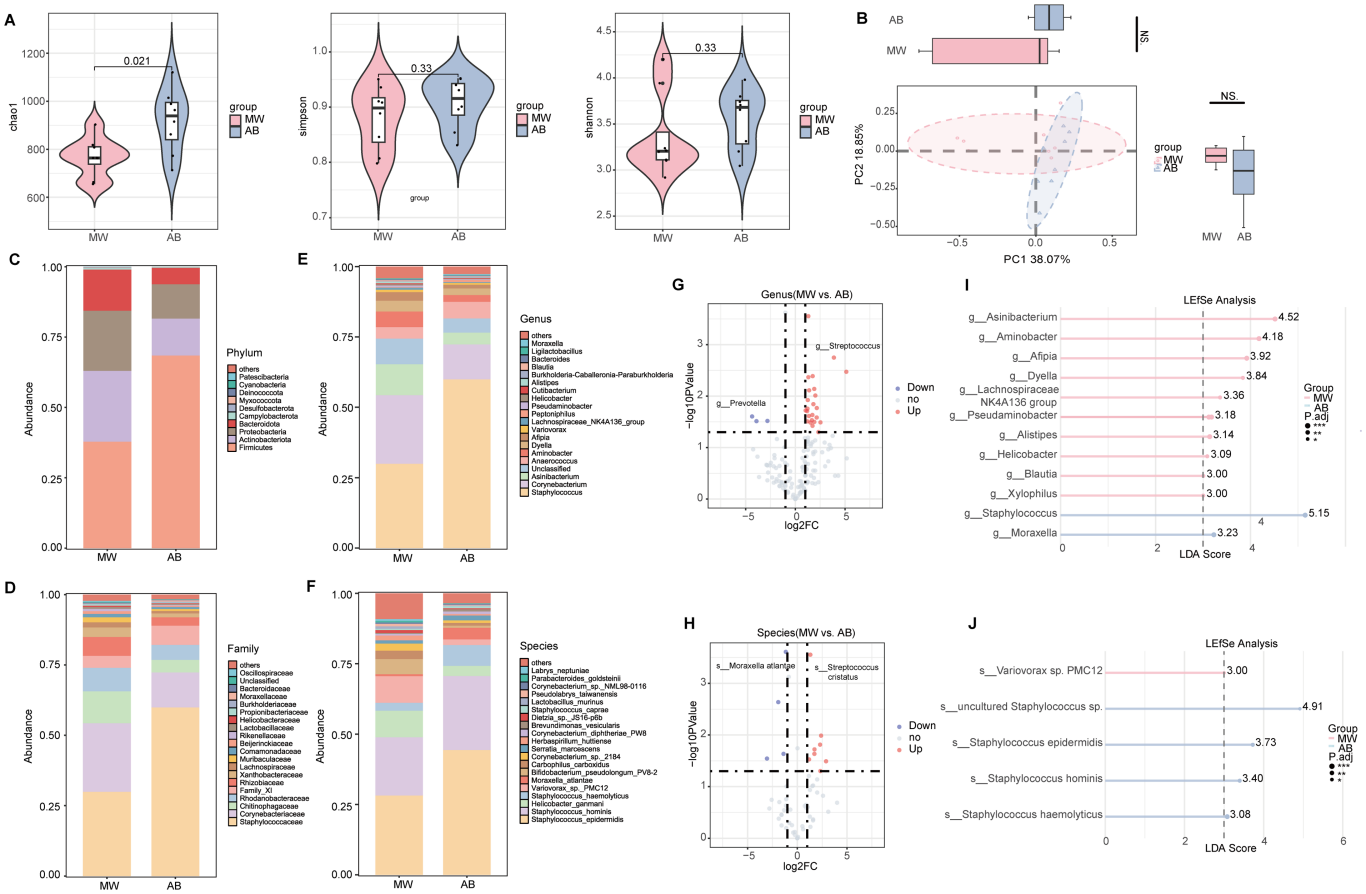
Axillary microbiota profiles of axillary bromhidrosis patients before and after microwave therapy. **(A)** α-diversity indices (Chao1, Simpson, Shannon). **(B)** Principal coordinate analysis (PCoA). **(C–F)** Taxonomic composition at the phylum, family, genus, and species levels. **(G)** Volcano plot of differential genera. **(H)** Volcano plot of differential species. **(I)** LEfSe analysis at the genus level. **(J)** LEfSe analysis at the species level. *FDR < 0.05, **FDR < 0.01, ***FDR < 0.001 indicate significant differences.

In the axillary microbiota, the MW group showed a significant decrease in Chao1 (*p* < 0.05), and a non-significant decrease in Shannon and Simpson indices compared to the AB group (Figure 4A). PCoA analysis revealed significant differences in the overall microbiota structure between the groups (Figure 4B). At the phylum level, Actinobacteriota was slightly more abundant, and Firmicutes was significantly less abundant in the MW group (*p* < 0.01) (Figure 4C). At the family level, Corynebacterium was slightly more abundant, while Staphylococcaceae was significantly less abundant in the MW group (*p* < 0.01) (Figure 4D). At the genus level, *Corynebacteriaceae* was slightly more abundant, and *Staphylococcus* was significantly less abundant in the MW group (*p* < 0.01) (Figure 4E). At the species level, *Helicobacter ganmani* was slightly more abundant, while *Staphylococcus epidermidis* and *Staphylococcus hominis* were significantly less abundant in the MW group (*p* < 0.01) (Figure 4F). Volcano plots at the genus and species levels identified 27 and 12 differentially abundant taxa, respectively, with 3 genera and 4 species downregulated, and 24 genera and 8 species upregulated in the MW group (relative abundance > 0.1%, FDR < 0.05) (Figures 4G,H). LEfSe analysis showed that the MW group was enriched in *Asinibacterium*, *Aminobacter*, and *Afipia* at the genus level, and *Variovorax* sp. *PMC12* at the species level (LDA > 3, *p* < 0.05) (Figures 4I,J).

**Figure 5:**
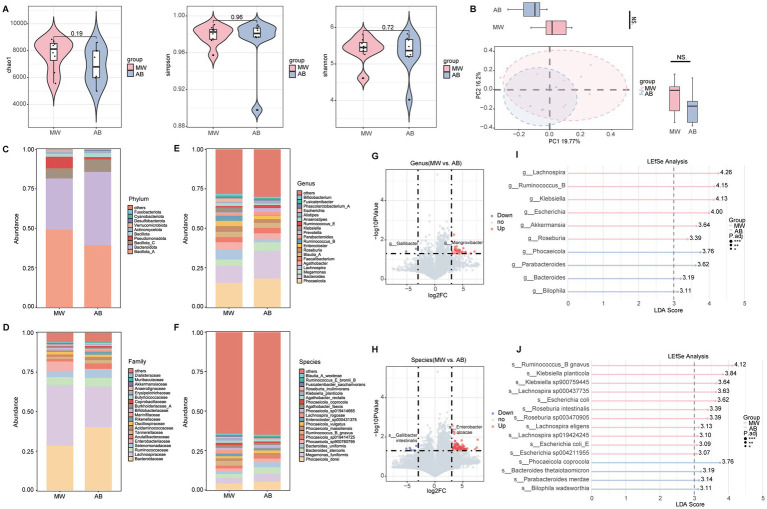
Gut microbiota profiles of axillary bromhidrosis patients before and after microwave therapy. **(A)** α-diversity indices (Chao1, Simpson, Shannon). **(B)** Principal coordinate analysis (PCoA). **(C–F)** Taxonomic composition at the phylum, family, genus, and species levels. **(G)** Volcano plot of differential genera. **(H)** Volcano plot of differential species. **(I)** LEfSe analysis at the genus level. **(J)** LEfSe analysis at the species level. *FDR < 0.05, **FDR < 0.01, ***FDR < 0.001 indicate significant differences.

In the gut microbiota, no significant differences in *α* and *β* diversity were found between the groups (Figures 5A,B). At the phylum level, Bacillota_A was slightly more abundant, and Bacteroidota was slightly less abundant in the MW group (Figure 5C). At the family level, Lachnospiraceae was significantly more abundant (*p* < 0.05), and Bacteroidaceae was slightly less abundant in the MW group (Figure 5D). At the genus level, *Lachnospira* was significantly more abundant (p < 0.05), while *Bacteroides* was slightly less abundant in the MW group (Figure 5E). At the species level, *Megamonas funiformis* and *Bacteroides stercoris* were slightly less abundant in the MW group (Figure 5F). Volcano plots at the genus and species levels identified 30 and 84 differentially abundant taxa, respectively, with 2 genera and 7 species downregulated, and 28 genera and 77 species upregulated in the MW group (relative abundance > 0.1%, FDR < 0.05) (Figures 5G,H). LEfSe analysis revealed that the MW group was enriched in *Lachnospira*, *Ruminococcus_B*, and *Klebsiella* at the genus level, and *Ruminococcus_B gnavus*, *Klebsiella planticola*, and *Escherichia coli* at the species level (LDA > 3, *p* < 0.05) (Figures 5I,J).

### KEGG functional and correlation analysis

**Figure 6:**
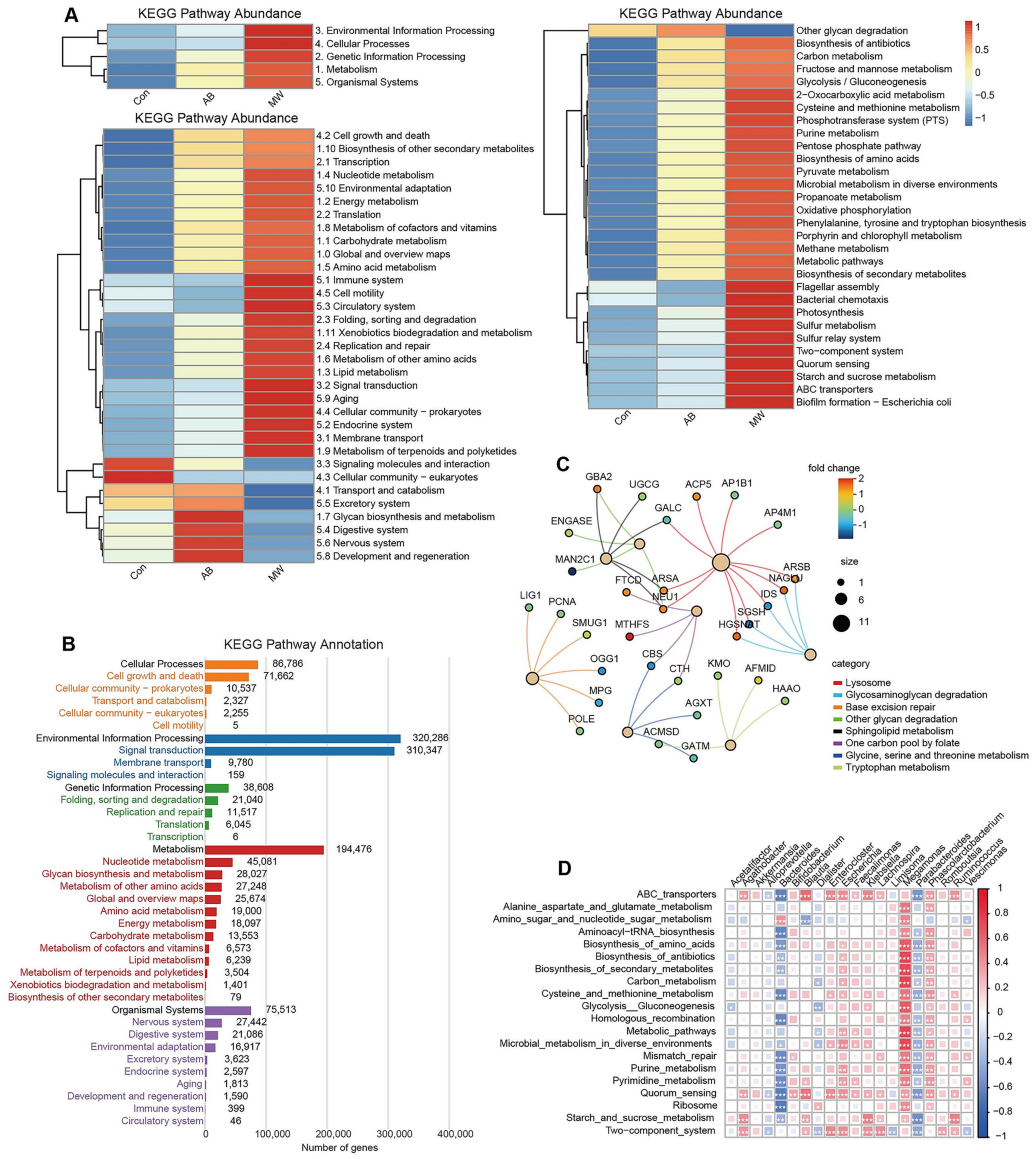
KEGG functional analysis and correlation analysis of gut microbiota. **(A)** Heatmaps showing correlations between groups and KEGG pathways at L1, L2, and L3 levels (red: positive correlation; blue: negative correlation; color intensity indicates correlation strength). **(B)** Bar plots of gene enrichment across KEGG pathways at L1 and L2 levels. **(C)** Gene–function correlation network; line colors indicate functional categories; node colors indicate fold change (FC); node size reflects the number of correlated genes. **(D)** Heatmap of correlations between genera and KEGG functional pathways (red: positive; blue: negative). ^*^p < 0.05, ^**^p < 0.01, ^***^p < 0.001 indicate statistical significance.

To explore the biological significance of gut microbes, KEGG pathway enrichment and correlation analysis were conducted. Pathway mapping was performed using the KEGG database. At the L1 level, the differential pathways mainly involved five major categories, including Metabolism and Genetic Information Processing. At the L2 level, key categories related to axillary bromhidrosis, such as Cell Growth and Death, Immune System, Lipid Metabolism, and Carbohydrate Metabolism, were highlighted. At the L3 level, pathways related to Sulfur Metabolism, Carbon Metabolism, Fructose/Mannose Metabolism, and Microbial Metabolism in Diverse Environments were focused on (Figure 6A). KEGG enrichment analysis revealed that many genes were enriched in pathways like Cell Growth and Death, Nucleotide Metabolism, and Amino Acid Metabolism, suggesting a close relationship between microbial communities and axillary bromhidrosis (Figure 6B). Correlation analysis identified genes such as MTHFS, FTCD, ARSA, NEU1, CBS, and NAGLU were highly correlated with the top 8 pathways, offering potential targets for further mechanistic studies (Figure 6C). Finally, correlation analysis revealed that genera like *Megamonas*, *Phascolarctobacterium* were significantly positively correlated with pathways like Cysteine and Methionine Metabolism, Quorum Sensing, and Microbial Metabolism in Diverse Environments, while *Bacteroides* and *Parabacteroides* showed the opposite pattern (*p* < 0.001) (Figure 6D).

## Discussion

Axillary bromhidrosis is a common condition characterized by abnormal body odor in the axillary region, closely linked to an imbalance in the skin microbiota. The gut-skin axis provides a theoretical basis for exploring the relationship between gut microbiota and axillary bromhidrosis. In this study, we used 16S rRNA sequencing and metagenomic analysis to identify axillary and gut microbiota associated with axillary bromhidrosis, and to further explore their potential correlation. Our results not only confirmed significant differences in axillary and gut microbiota composition between axillary bromhidrosis patients and healthy controls, but also revealed a clear correlation between the two communities. Furthermore, microwave therapy significantly altered axillary microbiota composition, reducing axillary odor intensity. These findings provide new insights into the microbial basis of axillary bromhidrosis and offer a novel direction for future microbiota-based systemic interventions.

We first explored the changes in the axillary microbiota between axillary bromhidrosis patients and healthy controls. The results revealed significant alterations in axillary microbiota composition and structure in patients, including increased abundance of *Staphylococcus* and related species. *Staphylococcus epidermidis*, one of the most common skin commensals, usually helps maintain skin health. However, when it overgrows in high sweat regions, such as the axilla, it metabolizes sebum and sweat to produce volatile organic compounds, leading to the development of axillary odor, especially acidic smells (Lam et al., 2018). *Staphylococcus hominis* and *Staphylococcus haemolyticus* are considered key odor-causing bacteria, efficiently metabolizing odorless precursors (e.g., Cys-Gly-3M3SH) secreted by apocrine glands. This process generates sulfur-containing volatile compounds (e.g., 3-Methyl-3-sulfanylhexan-1-ol), which contribute to the foul odor (Bawdon et al., 2015). Next, we investigated changes in the gut microbiota between axillary bromhidrosis patients and healthy controls. Although no significant changes were observed in the gut microbiota composition, shifts in key microbial species may influence axillary odor through metabolic and immune regulation. LEfSe analysis identified several species enriched in axillary bromhidrosis patients, such as *Bacteroides stercoris*, *Phocaeicola massiliensis*, *Phascolarctobacterium faecium*, and *Phocaeicola vulgatus*, which are known SCFAs producers. SCFAs can enter the bloodstream, affecting systemic metabolism and sebum secretion. They may be further converted into volatile sulfur compounds or isovaleric acid by skin microbiota, although direct experimental or clinical evidence for this relationship is still lacking (Gabashvili, 2020).

In the axillary microbiota correlation analysis, we found that *Lactobacillus* was significantly negatively correlated with odor-causing bacteria such as *Finegoldia*, *Anaerococcus*, and *Peptoniphilus*. Although *Lactobacillus* is present at low abundance in the axilla, several clinical studies have shown its therapeutic potential in reducing axillary odor. For instance, Li et al. reported that *Lactobacillus bulgaricus* treatment in axillary bromhidrosis patients reduced *Corynebacterium* abundance and odor scores, suggesting that *Lactobacillus bulgaricus* can suppress odor-causing bacteria and restore axillary microbiotal homeostasis (Li et al., 2022). These findings support the use of local probiotics can help suppress anaerobic cocci and reduce odor intensity. We also observed a positive correlation between *Staphylococcus haemolyticus* and *Serratia marcescens*. While *Serratia marcescens* is not a primary odor-causing bacterium, both species may coexist in the moist axillary environment through symbiotic biofilm formation. Interestingly, *Corynebacterium jeikeium* K411, a key odor-causing bacterium, produces strong-smelling volatile sulfur compounds by metabolizing sebum (Troccaz et al., 2015). The negative correlation between *Corynebacterium* and *Staphylococcus* may reflect competitive interactions in resource use or niche differentiation. In the gut microbiota correlation analysis, we found that genera positively correlated with *Blautia*, such as *Roseburia*, *Faecalimonas*, and *Enterocloster*, are dominant SCFAs producers (Fusco et al., 2023). Specifically, *Blautia* and *Roseburia* are major butyrate producers (Wu et al., 2022). *Escherichia coli*, a common anaerobic bacterium, ferments to produce gasses and SCFAs, and show positive correlation with *Anaerostipes hadrus*, another key butyrate producer (Herold et al., 2025). Correlation analysis between the axillary and gut microbiota revealed that odor-causing genera in the axilla, such as *Staphylococcus*, *Peptoniphilus*, and *Anaerococcus* (Okamoto et al., 2018), were positively correlated with gut genera like *Roseburia*, *Blautia*, and *Clostridium*, which produce SCFAs, particularly butyrate. SCFAs produced in the gut can enter systemic circulation and may be secreted into axillary sweat by sweat gland epithelial cells. These compounds can then converted by anaerobic bacteria into volatile odor molecules, such as sulfur compounds, contributing to axillary odor intensity (James et al., 2013). Gut dysbiosis may increase SCFAs levels, promoting axillary odor-causing bacteria proliferation. Conversely, high concentrations of odor metabolites in the axilla may influence gut microbiota metabolism (De Pessemier et al., 2021).

Interestingly, most gut genera positively correlated with axillary odor-causing bacteria are major butyrate producers. Butyrate, though beneficial for gut health, has a strong, unpleasant odor similar to rancid butter (Hays et al., 2024; Zhang et al., 2024; Yang et al., 2022). *In vitro* studies by Traisaeng et al. (2019) found that *Staphylococcus epidermidis* can produce butyrate through glycerol fermentation, inhibiting *Staphylococcus aureus* growth. However, the strong odor of butyrate limits its clinical application. Unfortunately, no studies have directly correlated butyrate levels in the blood or stool of axillary bromhidrosis patients with the chemical composition of axillary sweat or the intensity of the odor. Several studies have shown that fecal microbiota transplantation (FMT) can enrich butyrate-producing bacteria (including *Erysipelotrichaceae*, *Lactobacillaceae*, and *Eubacteriaceae*), thereby regulating gut microbiota stability and promoting the recovery of inflammation in dermatitis mice (Jiang et al., 2023). In pemphigus patients, reduced gut microbiota diversity and butyrate-producing bacteria are closely linked with clinical indicators (e.g., Dsg1/3 and PDAI), suggesting the potential impact of butyrate-producing bacteria on skin diseases (Guo et al., 2023). Trompette et al. (2022) demonstrated that gut-derived SCFAs, particularly butyrate, can enhance skin barrier function by modulating mitochondrial metabolism and the production of key structural components in epidermal keratinocytes, which is critical for maintaining cutaneous immune homeostasis. Recent studies have shown that gut-derived butyrate can modulate immune cell differentiation, such as enhancing stem-like tumor-specific CD8^+^ T cells in melanoma via FOXO1-driven programs. Although the mechanism in axillary bromhidrosis is likely distinct, the shared involvement of butyrate-producing microbiota suggests that SCFAs may play a modulatory role in skin-associated microbial-host interactions (Bachem et al., 2025). To date, there is no direct evidence supporting that gut-derived butyrate can substantially affect the axillary microbiota or sweat composition through the bloodstream or other pathways, thereby altering body odor. Therefore, more rigorous clinical and basic research is urgently needed to fill this significant gap in evidence. Additionally, we found that *Staphylococcus epidermidis* and other *Staphylococcus* species were positively correlated with SCFAs producers like *Bacteroides stercoris*, *Escherichia coli*, and *Bifidobacterium pseudocatenulatum*, while negatively correlated with *Alistipes onderdonkii* and *Lachnospira eligens*. *Alistipes onderdonkii*, an anti-inflammatory strain, may suppress axillary odor by reducing systemic inflammation, modulating metabolites, and regulating gut immune responses (Parker et al., 2020; Zhong et al., 2024). These findings suggest that axillary bromhidrosis is influenced not only by local axillary bacteria but also by the metabolic activities of the gut microbiota. Future research should focus on elucidating the molecular mechanisms of the gut-skin metabolic pathways, providing experimental evidence for targeted interventions in axillary bromhidrosis, such as modulating gut butyrate producers or locally inhibiting anaerobic odor-causing bacteria.

Next, we investigated the effects of microwave therapy on the axillary and gut microbiota in axillary bromhidrosis patients. The results showed that microwave therapy decreased the *α*-diversity of axillary microbiota composition, suggesting that microwave therapy may disrupt skin ecological niches (e.g., sweat glands, hair follicles), thereby reducing colonizable bacterial species. However, the abundance of *Corynebacterium* increased post-treatment. *Corynebacterium* is one of the key genera responsible for converting odorless steroid precursors in sweat into strongly odorous compounds like 16-androstenes (Chang and Wang, 2023). Microwave therapy damages apocrine glands and hair follicles through volumetric heating, significantly reducing sweat secretion; nevertheless, the residual axillary environment (low moisture, increased temperature) may provide a relative advantage to *Corynebacterium*, which is more resilient to dryness (Zhang et al., 2022). Meanwhile, moisture-dependent bacteria like *Staphylococcus* showed a significant decrease, suggesting that microwave therapy may alleviate axillary odor by inhibiting these odor-causing bacteria. LEfSe analysis further revealed the emergence of environmentally adapted bacteria (e.g., *Asinibacterium*, *Aminobacter*), which may be more competitive in dry, low-oxygen environments, indicating that microwave therapy altered the ecological niche structure of the microbiota. If mild odor or local irritation persists clinically, further microbiota modulation (e.g., probiotics, local antimicrobial treatments) could optimize therapeutic outcomes. In the gut microbiota analysis, microwave therapy did not significantly alter the richness or structure, consistent with the physical properties of microwave energy being confined to skin tissue. The mild upregulation of *Firmicutes* and enrichment of some metabolism-related bacteria post-treatment may reflect slight modulation of systemic metabolism or immune status. LEfSe analysis showed that the enriched *Lachnospira* and *Ruminococcus_B* were positively correlated with SCFAs production, suggesting that microwave therapy may indirectly promote the growth of beneficial bacteria through the gut-skin axis. However, the enrichment of *Klebsiella* and *Escherichia coli* requires caution, as these facultative pathogens may proliferate under immunosuppression or compromised intestinal barrier function (Stanton and Wyres, 2024; Hu and Torres, 2015). Given the limited sample size, clinical risks cannot be conclusively determined. Overall, our findings suggest that microwave therapy is not only a local physical deodorizing method, but may also facilitate systemic microbiota modulation through the gut-skin axis. Further large-scale, long-term studies and functional validation are needed to provide a solid foundation for understanding the mechanisms of microwave therapy and for developing personalized treatment plans.

To explore the biological significance of the gut microbiota, we performed KEGG pathway enrichment and correlation analysis. At the L1 level, microwave therapy altered the overall metabolic activity of the gut microbiota. At the L2 level, pathways such as Lipid Metabolism, which is related to sebum secretion and fatty acids metabolism generating volatile odor molecules, and Carbohydrate Metabolism, which provides bacterial substrates and affects the types and amounts of metabolites, were found to be potentially linked to axillary bromhidrosis (James et al., 2013; James et al., 2004). Furthermore, Glycan Biosynthesis and Metabolism decreased post-treatment, suggesting that reduced glycosylation levels might weaken bacterial adhesion (Moonens and Remaut, 2017). At the L3 level, pathways like Sulfur Metabolism and Carbon Metabolism, which are closely related to the production of odor components (e.g., sulfur compounds and volatile fatty acids) in axillary bromhidrosis. For example, Sulfur Metabolism directly influences the production of sulfur-containing odors, typically generated by bacteria like *Corynebacterium* (Natsch et al., 2004). KEGG enrichment analysis revealed that several genes were enriched in pathways such as Cell Growth and Death, Nucleotide Metabolism, and Amino Acid Metabolism. Notably, Amino Acid Metabolism plays a central role in axillary bromhidrosis, as most volatile odor molecules originate from bacterial enzymatic cleavage of amino acid-acyl precursors, including sulfur amino acids that produce sulfur compounds and other amino acid-derived amines (Natsch and Emter, 2020; Di Cicco et al., 2023). Gene pathway network analysis identified genes such as MTHFS, FTCD, and ARSA were highly correlated with key functions, forming the core of axillary bromhidrosis metabolism and providing a molecular foundation for further investigation. Genes like CBS (Cystathionine β-synthase) and ARSA (Arylsulfatase A) may be directly involved in the generation of sulfur-containing metabolites in axillary bromhidrosis, warranting further exploration of their roles (Morris et al., 2017). Additionally, genera like *Megamonas* and *Phascolarctobacterium* may contribute to odor production by metabolizing sulfur-containing compounds like cysteine and methionine, while *Bacteroides* and *Parabacteroides* may play a protective role by inhibiting these processes. These findings offer valuable insights into the relationship between the gut microbiota and axillary bromhidrosis, offering potential microbial targets for future intervention strategies.

However, our study has some limitations that require further validation. First, the relatively small sample size and gender imbalance may affect statistical power of the results and limit their generalizability. Future studies should increase the sample size and ensure a more balanced gender distribution to improve the reliability and external validity of the findings. Second, although we explored the effects of microwave therapy on the axillary and gut microbiota in axillary bromhidrosis patients, the underlying therapeutic mechanism remains unclear. Moreover, the small sample size of the microwave therapy group (*n* = 8) limited the statistical power and may have affected the reliability of the conclusions. Future research should expand the sample size to enhance statistical power, investigate how microwave therapy regulates the microbiota to alleviate axillary bromhidrosis and evaluate its potential benefits in long-term interventions. Third, the prediction of microbial metabolic pathways using 16S rRNA gene sequencing and tools such as PICRUSt2 has inherent limitations and cannot directly reflect actual metabolic changes. Future studies are planned to incorporate metabolomic analyses to directly measure metabolites associated with the axillary and gut microbiota, in order to validate the results of the predictive metabolic pathway analysis. Lastly, our study mainly relied on observational data and did not conduct interventional experiments to establish causal relationships between gut microbiota and axillary bromhidrosis. Although our correlation analysis found a positive correlation between butyrate-producing bacteria in the gut and several axillary odor-causing bacteria, it remains unclear whether butyrate produced in the gut affects the axillary microbiota and regulates axillary bromhidrosis. In future studies, we plan to utilize twin/family studies or conduct genome-wide association studies (GWAS) and genotype stratification to control for genetic background. Systemic metabolite assessments (e.g., blood SCFAs, immune markers) and multivariate statistical models (e.g., partial correlation analysis, structural equation modeling) will be employed to control for these metabolic variables. Additionally, we will also investigate whether key butyrate receptors (e.g., GPR41 and GPR43) are expressed in human axillary sweat glands, eccrine glands, or keratinocytes. With sufficient experimental data, we plan to design a small-scale randomized controlled trial to assess the causal relationship between gut-derived butyrate and axillary bromhidrosis, using the changes in the volatile organic compound profile of axillary sweat and objective odor evaluation as endpoints.

## Conclusion

Axillary bromhidrosis remains an unresolved health issue, and understanding the relationship between the axillary and gut microbiota is essential for revealing its pathogenesis and identifying potential microbial therapies. Our results show a significant imbalance in the axillary microbiota of patients, particularly with the enrichment of *Staphylococcus* and related species, which are key contributors to the condition. Furthermore, while the gut microbiota composition showed no significant changes, but LEfSe analysis revealed SCFAs-producing bacteria was elevated. This suggests that the gut microbiota may influence the skin microenvironment through metabolic products, indirectly promoting axillary bromhidrosis. Importantly, we are the first to explore the correlation between the axillary and gut microbiota, revealing a positive correlation between butyrate-producing bacteria in the gut and several axillary odor-causing bacteria. This indicates that both may form a bidirectional microbial network through gut-derived metabolites (especially butyrate) and immune modulation. However, this causal relationship still needs to be confirmed through clinical cohort studies and interventional experiments, particularly considering the regulatory effects of host genetics and metabolic status on both the axillary and gut microbiota. Additionally, we found that microwave therapy may alleviate axillary bromhidrosis by altering the axillary microbiota structure and inhibiting the proliferation of odor-causing bacteria, though it has minimal impact on the gut microbiota. This study not only provides new insights into the microbial basis of axillary bromhidrosis, but also expands the theoretical framework of the gut-skin axis, offering new directions for future axillary bromhidrosis treatments based on systemic microbial regulation.

## Data Availability

All sequencing data generated in this study have been deposited in the NCBI database under accession number PRJNA1456328.

## Supplementary material

SUPPLEMENTARY FIGURE S1 Correlation analysis of differential axillary microbiota between axillary bromhidrosis patients and healthy controls. **(A)** Genus-level correlations. **(B)** Species-level correlations.

SUPPLEMENTARY FIGURE S2 Correlation analysis of differential gut microbiota between axillary bromhidrosis patients and healthy controls. **(A)** Genus-level correlations. **(B)** Species-level correlations.
