# Supplementary figures and images for: Axillary and gut microbiota characteristics in axillary bromhidrosis patients and the effect of microwave therapy: a case-control study
Source: Front Microbiol. 2026 May 5;17:1769465. doi: 10.3389/fmicb.2026.1769465 (PMC13183811; doi:10.3389/fmicb.2026.1769465)

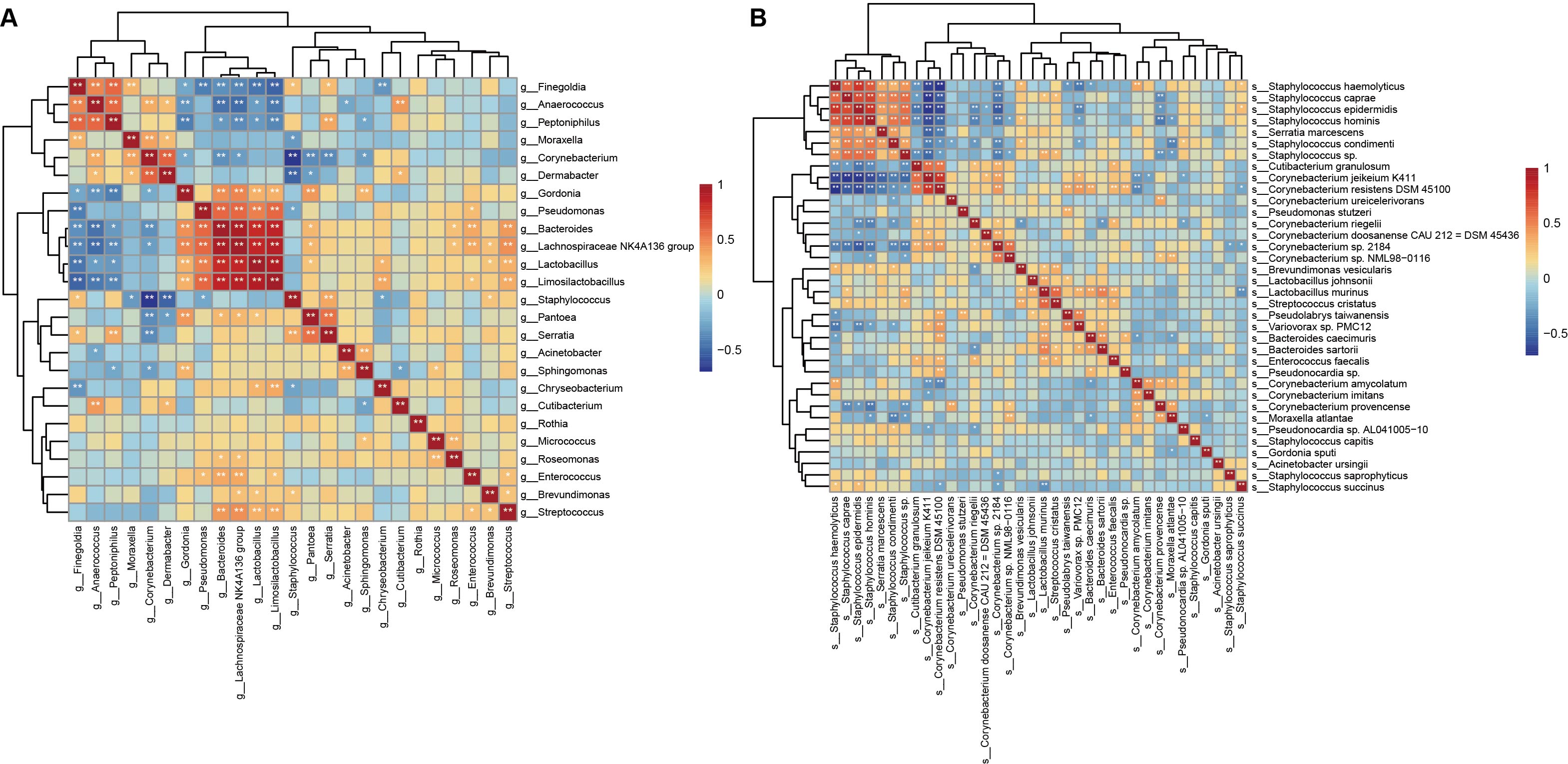

Supplement: SUPPLEMENTARY FIGURE S1 — Correlation analysis of differential axillary microbiota between axillary bromhidrosis patients and healthy controls. (A) Genus-level correlations. (B) Species-level correlations. [file Image_1.JPEG]

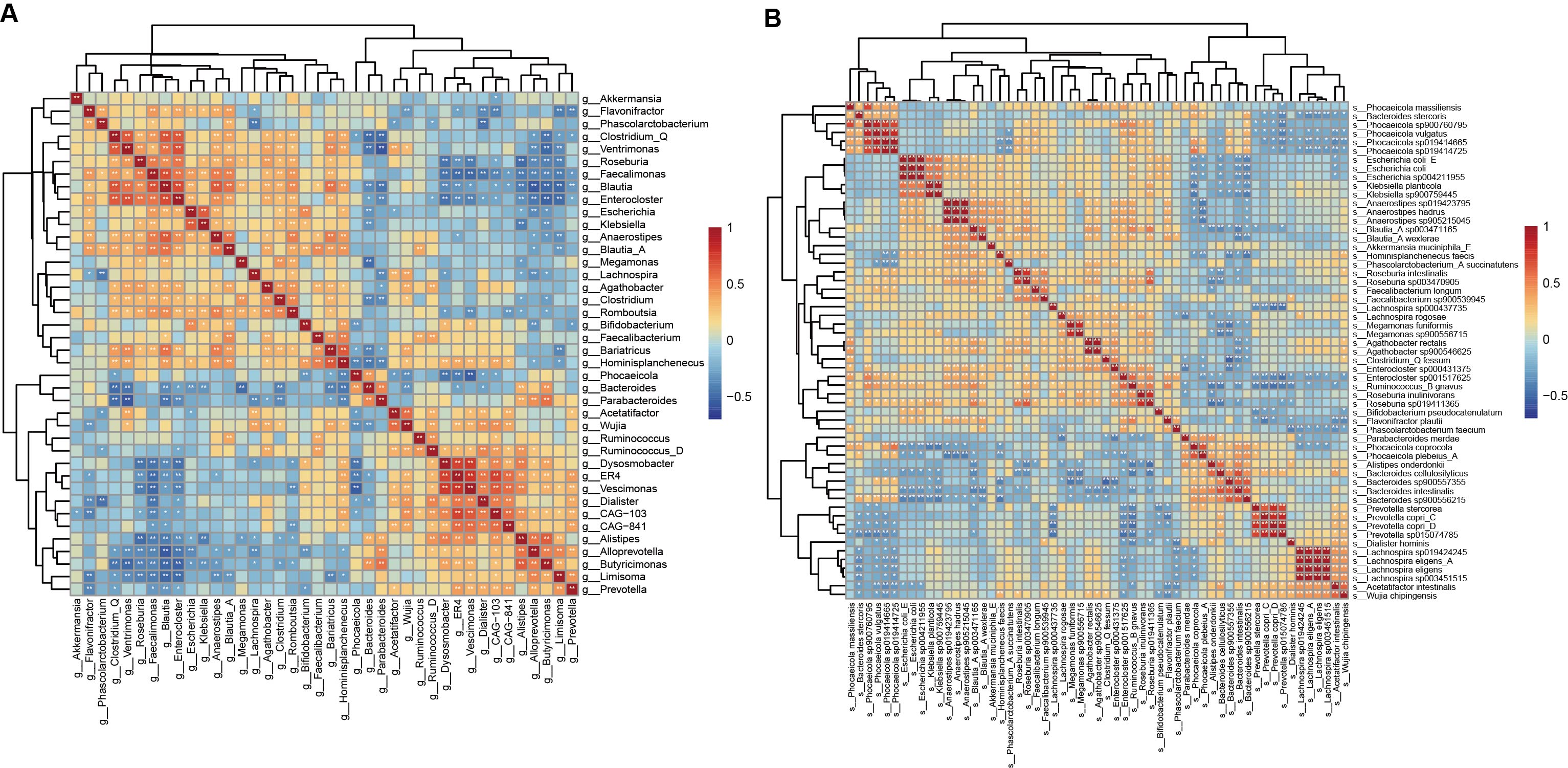

Supplement: SUPPLEMENTARY FIGURE S2 — Correlation analysis of differential gut microbiota between axillary bromhidrosis patients and healthy controls. (A) Genus-level correlations. (B) Species-level correlations. [file Image_2.JPEG]
